# The complete chloroplast genome of *Lonicera similis* Hemsl. and its phylogenetic analysis

**DOI:** 10.1080/23802359.2021.1987167

**Published:** 2021-10-07

**Authors:** ShaoXiong Wu, ChunYan Jiang, XiaYu Feng, ChenJu Yang, ZhengWen Yu

**Affiliations:** School of Life Sciences, Guizhou Normal University, Guiyang, China

**Keywords:** *Lonicera similis* Hemsl., cp genome, phylogenetic, genetic study

## Abstract

*Lonicera similis* Hemsl. belongs to the Caprifoliaceae family and used as a substitute for ‘jin yin hua’. Recent years, it demonstrates great economic value because of its rich chemical composition. However, the phylogenetic relationship between *L. similis* and other family members remains unclear. In this paper, we assembled the cp genome of *L. similis* using the high-throughput Illumina pair-end sequencing data. The circular cp genome was 155,207 bp in size, including a large single-copy (LSC) region of 88994 bp and a small single-copy (SSC) region of 18,633 bp, which were separated by two inverted repeat (IR) regions (23,790 bp each). A total of 121 genes were predicted, including eight ribosomal RNAs (rRNAs), 36 transfer RNAs (tRNAs), and 77 protein-coding genes (PCGs). In addition, the result of phylogenetic analysis indicated that *L. similis* formed a close relationship from another congeneric species (*Lonicera confusa*). This study provides helpful information for future genetic study of *L. similis.*

*Lonicera japonica* Thunb is a kind of well-known traditional Chinese medicine and has a common known name as ‘jin yin hua’ in Chinese. It has been used to treat fever, eliminating inflammation and antibacterial (Weng et al. 2011; Hu et al. 2012; Zeng et al. 2020). *Lonicera similis* Hemsl. is mainly founded in south shaanxi, south gansu and other regions in China, which belongs to the Caprifoliaceae family and has the same function with Lonicerae Japonicae Flos in modern pharmacological studies (National Pharmacopeia Committee 2015). Therefore, it is used as a substitute for ‘jin yin hua’ in mountainous areas of southwest China (Zhang et al. 2018). At present, the research on chemical composition has been in-depth, involving organic acid, flavonoids, saponins, volatile oil and so on (Lv et al. 2012). Among them, chlorogenic acid is the most important secondary metabolites of medicinal materials of honeysuckle (Cai et al. 2020) and its content in the *L. similis* has been proved higher than the current pharmacopeia standards(Ma et al. 2007). Although *L. similis* contains lots of valuable information and economic benefits through chemical composition research, there has rarely been any report for a genetic study of the plant. Therefore, the complete chloroplast (cp) with many advantages has become our ideal tool for phylogeny research (Dong et al. 2014; Saina et al. 2018). In this paper, the cp genome of *L.similis* Hemsl. has been assembled and determined. The study will be a helpful resource for future genetic research and determining phylogenetic relationships.

Healthy and fresh leaf samples were picked from Dangwu Town, Huaxi District, Guiyang City, Guizhou Province, China (26°23′34.49″N, 106°35′56.89″E, 1,158 m above sea level). The plants were identified using a species identification bench mark set up by Dr. Chunyan Han, Kunming Caizhi Biotechnology Co. Ltd, Kunming, Yunnan, China. A voucher specimen was deposited at a local herbarium of School of Life Sciences, Guizhou Normal University (contact person named Wenqing He and his e-mail is 2829501932@qq.com) with accession numbers GZNUYZW202101002. The total genomic DNA (No. YX20210115901) was extracted using E.Z.N.A Plant DNA kit (FEIYANG, Guangzhou, China) and stored in the biochemical laboratory (room number: 1403) of School of Life Science, Guizhou Normal University. A total amount of 1000 ng DNA per sample was used as input material for the DNA sample preparations. Sequencing libraries were generated using NEB NextV RUltra DNA Library Prep Kit for IlluminaV R (NEB, Ipswich, MA). Total DNA was used to generate libraries with an average insert size of 400 bp. The library preparations were sequenced on an Illumina platform and 150 bp paired-end reads were generated.The program GetOrganelle will be used to assemble the filtered reads with *Lonicera japonica* as the initial reference genome(GenBank accession number: MH028738) and the assembled cp genome will be annotated using the online software GeSeq (Michael et al. 2017; Jin et al. 2020). Finally, the precisely annotated complete cp genome was submitted to GenBank with accession number MZ241297.

The length of the complete cp genome sequence of *L. similis* was 155,207 bp. It shows a single-circular molecule with a four-segment structure, consisting of a large single-copy (LSC, 88,994 bp) region, a small single-copy (SSC, 18,633 bp) region, and two inverted repeat (IRA and IRB) regions of 23,790 bp each. A total of 121 genes were predicted, including 77 protein-coding genes (PCGs), eight ribosomal RNA (rRNA) genes, and 36 transfer RNA (tRNA) genes. Among these assembled genes, all rRNAs, four PCGs (*ycf2, rps7, ndhB, rps12*) and seven tRNAs *(trnN-GUU, trnR-ACG, trnA-UGC, trnI-GAU, trnV-GAC, trnL-CAA, and trnI-CAU)* were with double copies. Intron-exon analysis showed the majority (103 genes, 85%) genes with no introns, whereas 18 genes (15%) contain introns.

To make further research on the cp genome of *L. similis*, we choose mega7.0 software for alignment (https://www.megasoftware.net/). There were 21 cp genome sequences of Caprifoliaceae family (13 *Lonicera* species, three species from *Patrinia* genus, two species from *Dipelta* genus and the remaining of three species are from *Heptacodium, Triosteum and Weigela*) downloaded from GenBank to construct the phylogenetic tree through maximum-likelihood (ML) analysis. The ML tree based on GTR + gamma + I model was performed using RAxML (Version 8.0.19) with 1000 bootstrap replicates (Stamatakis 2014). We analyzed from the phylogenetic tree that *L. similis* belongs to genus *Lonicera* (Figure 1) and formed a nearly clade from *L. confusa.* It shows their relationship is the closest, and the support rate is 100%. Although *L. japonica.* is separate from *L. similis,* they still get a strong sister relationship. It also indicates we should make more comparative research between *L. similis* and other two species (*L. confusa* and *L. japonica)* to discover the potential economic value of *L. similis.*

**Figure 1.**
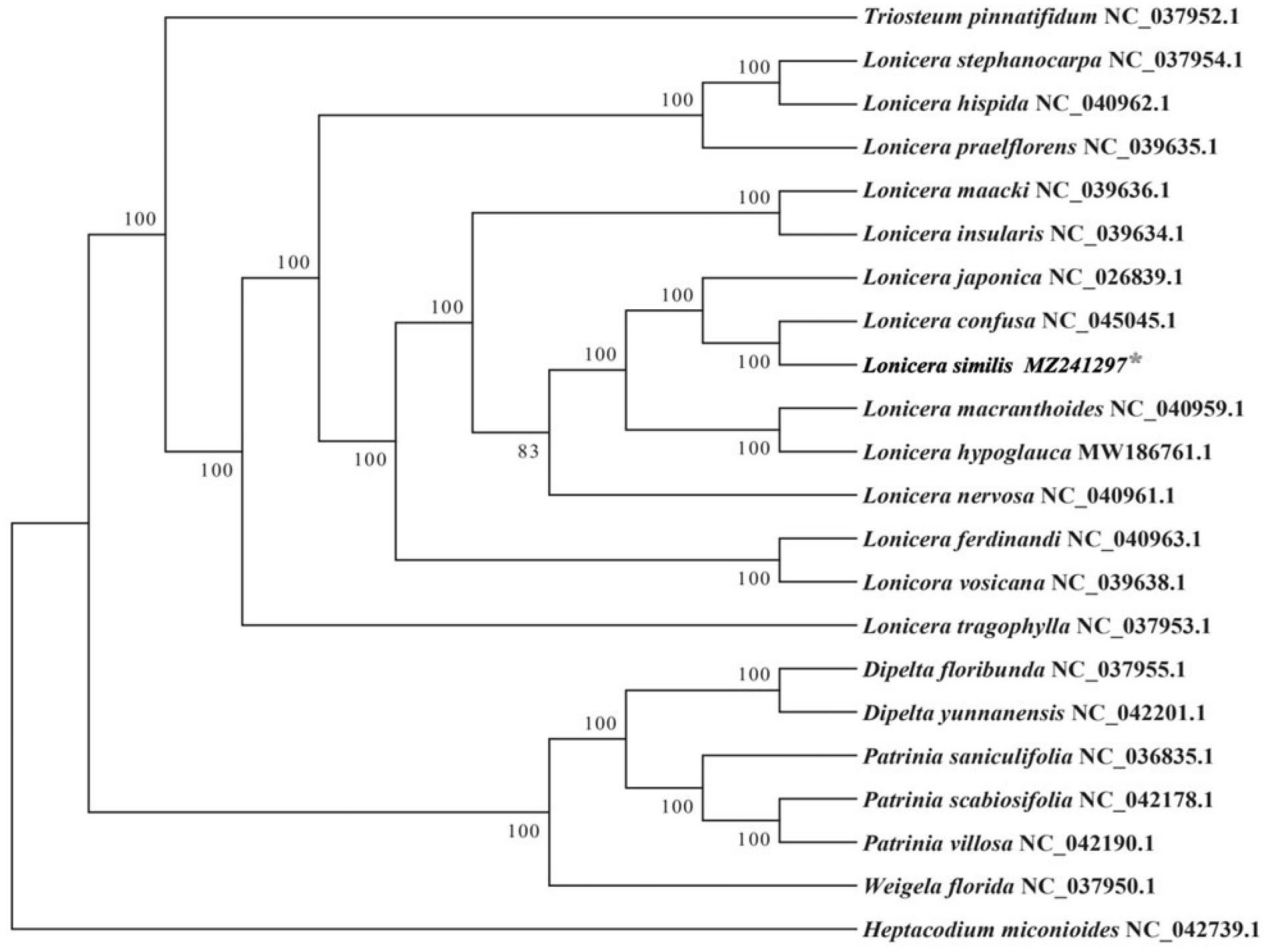
The complete cp genome sequences of 21 species from the Caprifoliaceae family are shown on this Maximum-likelihood tree. The numbers next to the nodes are bootstrap support values based on 1000 replicates. GenBank accession numbers are marked after the species name.

## Data Availability

The annotated chloroplast genome data that support the findings of this study are openly available in GenBank of NCBI at https://www.ncbi.nlm.nih.gov under the accession number MZ241297. The associated BioProject, SRA, and Bio-Sample numbers are PRJNA758746, SRR15665302, and SAMN21033719 respectively.
